# Standardized approach for four-fenestrated physician-modified endograft to treat complex abdominal aortic aneurysms using Valiant Captivia

**DOI:** 10.1016/j.jvscit.2024.101491

**Published:** 2024-03-26

**Authors:** Michele Piazza, Francesco Squizzato, Andrea Spertino, Franco Grego, Michele Antonello

**Affiliations:** Vascular and Endovascular Surgery Division, Department of Cardiac, Thoracic, Vascular Sciences and Public Health, University of Padua School of Medicine, Padua, Italy

**Keywords:** Aorta, Abdominal aortic aneurysm, Endograft, Physician modified endograft, FEVAR, Urgent repair

## Abstract

We describe the feasibility and safety of a standardized approach for four-fenestrated physician-modified endograft (PMEG) placement to treat complex abdominal aortic aneurysms using the Valiant Captivia platform (Medtronic). The standardization is based on specific selection criteria for anatomical feasibility, measurement method, and modification technique of a single endograft type. Six cases (two juxtarenal, two pararenal, and two type IV thoracoabdominal aneurysms) were treated, with 24 target vessels incorporated with fenestrations. Four cases were treated in an urgent setting and two were elective. The time modification required was 121 ± 18 minutes. Technical success was 100%, with no mortality or complications at 30 days. Postoperative computed tomography at 3 months demonstrated complete aneurysm exclusion, without a type I or III endoleak, no main graft- or fenestration-related loss of integrity, and no target vessel misalignment or stent fracture. The present standardized approach seems safe and feasible and might represent an initial benchmark for comparison with future studies.

Abdominal aortic aneurysms (AAAs) without an adequate proximal neck length for safe sealing and fixation with standard endovascular aneurysm repair (EVAR) might benefit from complex endovascular repair. The most common are short neck AAAs, juxtarenal AAAs, pararenal AAAs, and type IV thoracoabdominal aneurysms (TAAAs), where incorporation of renal and visceral vessels is required to obtain adequate proximal sealing. Several endovascular options are available, with the most effective represented by incorporation of target vessels by custom-made or off-the-shelf fenestrated/branched endografts.^1^

Custom-made devices have shown excellent early and long-term outcomes^2^, ^3^, ^4^; however, due to the manufacturing time required (up to 3 months), they can be used only for elective cases. For urgent cases, readily available off-the-shelf devices can be used; however, their anatomical feasibility is not exhaustive and might require long segment aortic coverage above the celiac trunk.^5^ Parallel aortic grafts with chimneys or periscopes have been proposed as timely alternatives; however, gutter endoleaks and the lack of proximal sealing are serious concerns for these techniques, especially for those cases for which more than two target vessels must be incorporated.^6^^,^^7^

For patients presenting with a large aneurysm, symptomatic patients, and those requiring urgent repair, the use of a physician-modified endograft (PMEG) represents a valid option. A PMEG allows for rapid customization of a standard abdominal or thoracic device with the creation of fenestrations, scallops, or branches, based on the aneurysm’s anatomical extent and target vessel position.

However, these techniques demand a high level of skills and experience. A recent review also showed that in the literature there is extreme variability on the indications for treatment, type of aneurysm and extension, and type of graft and method of modification, limiting the possibility to adequately analyze outcomes or perform comparisons between different techniques.^8^

We describe our initial experience on the safety and feasibility of a standardized approach with a PMEG, based on specific anatomical selection criteria, measurement method, and a standard technique of modification, using the Valiant Captivia thoracic endograft (Medtronic).

## How we do it

### Anatomical feasibility

We reserve this approach to AAAs with a >6-cm diameter and associated with clinical and anatomical features with a high risk of rupture (eg, saccular morphology, irregular thrombus, unclear outer wall identification, rapid growth), large AAAs (>7 cm), and symptomatic and urgent cases. We exclude emergent cases requiring immediate treatment because of hemodynamic instability or hemorrhagic shock. An internal hospital protocol was developed for these procedures (protocol no. 0002132).

This PMEG technique is performed for cases of complex AAAs, intended as short-neck AAAs (<5 mm), juxtarenal or pararenal AAAs, and type IV TAAAs. All these anatomical conformations have in common the possibility of being treated endovascularly using low supraceliac coverage (within 5 cm from the mid-celiac artery) as classified in the Society for Vascular Surgery (SVS) reporting standard.^9^ Anatomical feasibility is based on the presence of a proximal aortic neck above the mid-celiac trunk ≥25 mm long and between 18 mm and 35 mm in diameter. The neck must be “healthy,” with no parietal calcifications or thrombus and with a cylindrical shape. This is to allow the largest tapered Valiant graft (38 mm proximally to 34 mm distally) to fit the largest distal EVAR graft available (36 mm). The visceral aorta must be no larger than 40 mm in maximum diameter to decrease the distance between the target vessel ostium and graft fenestration.^10^^,^^11^ The mismatch in diameter between the supraceliac aorta and the pararenal aorta must be <30%, with a minimal vertical or lateral distance between fenestrations of 3 mm.

### Technique and procedure

**Fig 1:**
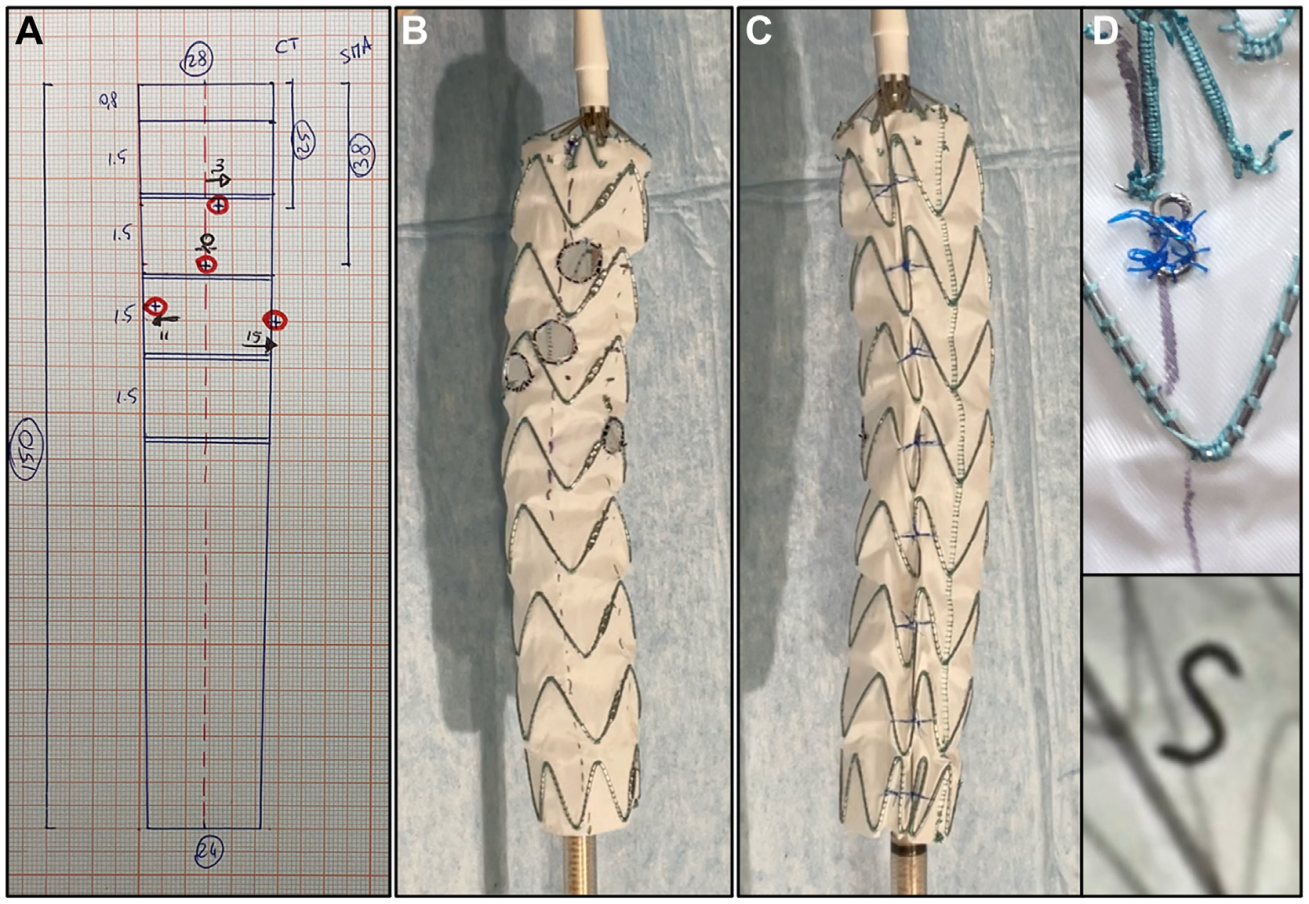
**A,** Technical sketch of the modified graft on graph paper. **B,** Anterior view of the physician-modified endograft (PMEG) after completion of the modification and before graft resheathing. **C,** Posterior view of the PMEG after completion of the modification and before graft resheathing. Note the diameter-constraining wire. **D,** Detail of the “S” marker positioned on the top of the graft on the anterior aspect to aid in anteroposterior orientation of the PMEG during implantation.

Preoperative planning includes measurement of the target vessel vertical and lateral positions using the centerline based on SVS standards.^9^ The measures are then transposed to graph paper for a technical draft, based on the fenestration allocation in relation to the desired graft diameter and length selected (Fig 1, *A*).

The graft is modified on a sterile back table in a hybrid operating room, while the patient is prepared for general anesthesia. A Valiant Captivia thoracic graft with free flow (with either a tubular or tapered shape) is fully deployed without releasing the free flow, which is maintained retained on its tip. Next, a vertical dotted line is drawn to mark the graft’s anterior 0°, and fabric holes are performed with ophthalmologic cautery in the desired position, taking care to not have graft struts within the fenestrations. The fenestrations must nicely rounded, ideally 6 × 6 mm for the renal vessels and 8 × 8 mm for the superior mesenteric artery and celiac trunk, and are reinforced with the tip of a 0.018-in. guidewire (V-18; Boston Scientific) through a continuous 5-0 nonabsorbable locking suture (Ti-Cron; Medtronic; Fig 1, *B*).

A posterior constraining wire is created to temporarily reduce the diameter of the graft by 20% to 30% to allow for partial rotation during the deployment phase. The back of a 0.018-in. guidewire is routed on the posterior aspect of the graft, passing within the external suture of the struts and, thus, not requiring fabric puncture (Fig 1, *C*). The Prolene suture used to reduce the diameter are tied around the stent struts and subsequently looped to the posterior wire. The four 8-shaped markers on the top of the graft are carefully removed with a scalpel. One of these is modified as an “S” and resutured on the anterior aspect at 0° position, with a 5-0 Prolene continuous suture. This “S” marker will allow for anteroposterior graft orientation (Fig 1, *D*). Finally, the graft is resheathed with help of a vessel loop (Supplementary Video).

**Fig 2:**
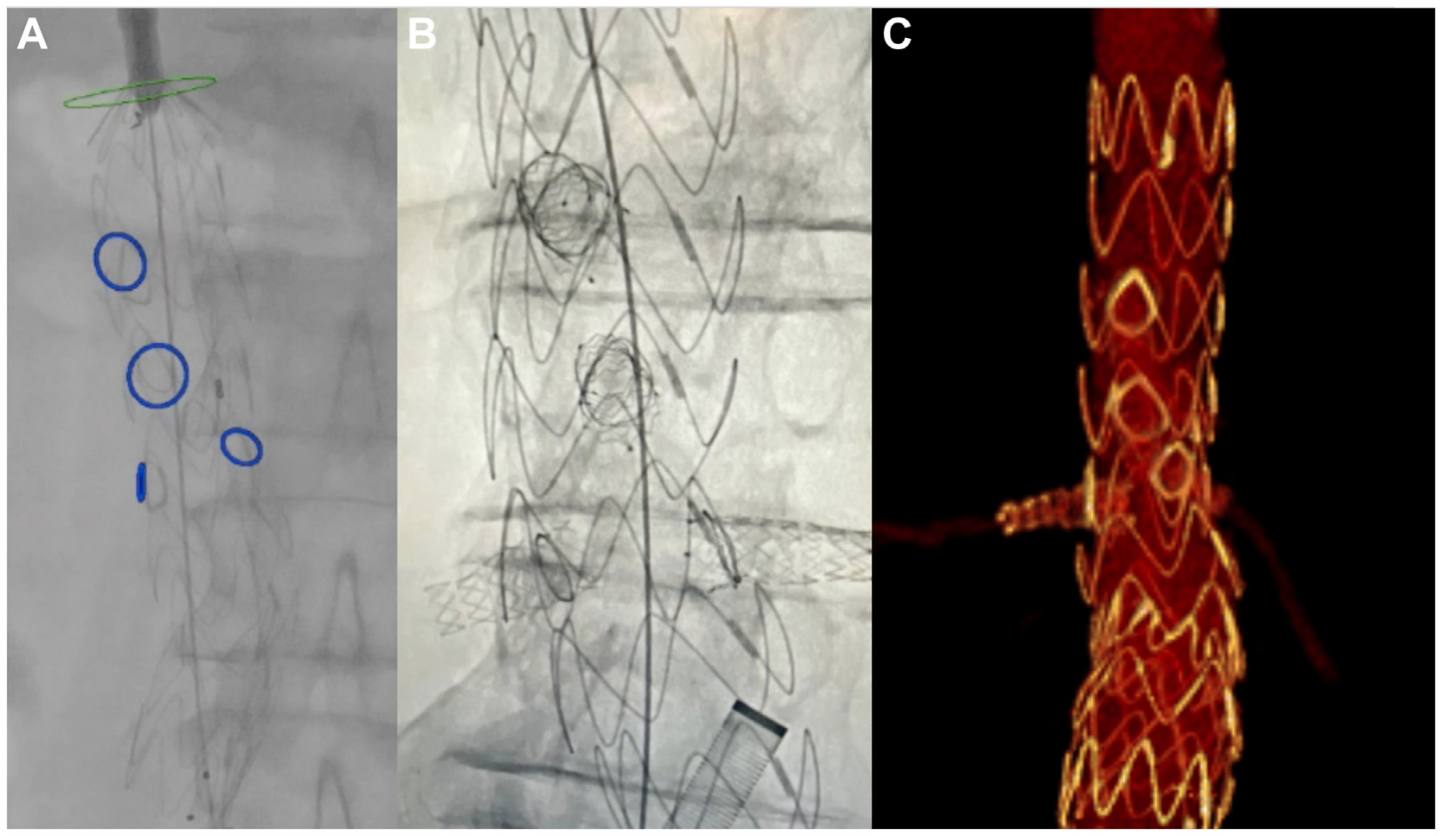
**A,** Intraoperative image showing physician-modified endograft (PMEG) deployment. Note the alignment between the fenestrations and overlay fusion rings indicating the origin of the target vessels. **B,** Intraoperative fluoroscopy image after PMEG deployment and positioning of the bridging stents. **C,** Three-dimensional image of the postoperative computed tomography angiogram.

Once the graft is ready, it is first oriented on the anteroposterior view outside the patient, verifying the correct “S” marker orientation, and then introduced via a femoral access and deployed under fusion imaging guidance, with the fenestrations at the level of the correspondent target vessel (Fig 2, *A*). When one half of the graft length is fully deployed with the fenestrations well expanded, the orientation can still be modified using the reducing tie to reach perfect fenestration alignment. Once the final desired position is reached, full deployment is completed. From the contralateral femoral access, the target vessels are cannulated, and the constraining wire is removed. At this point, the free flow is released and the procedure completed with bridging stent implantation as usual. A distal EVAR graft is implanted to complete AAA exclusion. Final angiography with associated cone-beam computed tomography (CT) is finally performed to assess technical success, implant integrity, and geometric conformation^10^^,^^12^ (Fig 2, *B*). CT angiography is performed immediately postoperatively and within 3 months for all cases to assess short-term safety and device integrity (Fig 2, *C*).

## Results

Six consecutive patients met the anatomical criteria and were treated during a 6-month period, with 24 incorporated target vessels. The clinical presentation, aneurysm anatomical characteristics, and type of graft used are reported in Table I, Table II.

**Table I:** Case series summary

Variable	Case 1	Case 2	Case 3	Case 4	Case 5	Case 6
Setting	Urgent	Urgent	Elective	Elective	Urgent	Urgent
SVS score	10	11	8	8	11	10
Aneurysm extent	Juxtarenal	Pararenal	Type IV	Type IV	Pararenal	Juxtarenal
Maximal anterior diameter, mm	68	65	90	78	62	78
Presentation	Lumbar pain	Abdominal pain	Asymptomatic	Asymptomatic	Abdominal pain	Lumbar pain
ICU stay, days	2	–	–	–	2	1
Length of stay (days)	3	3	2	5	4	4
MAE	–	–	–	–	–	–
Reintervention	–	–	–	–	–	–

*ICU*, Intensive care unit; *MAE*, major adverse events (within 30 days); *SVS*, Society for Vascular Surgery.

**Table II:** Anatomical and procedural details

Variable	Case 1	Case 2	Case 3	Case 4	Case 5	Case 6
Access	Percutaneous femoral	Percutaneous femoral	Percutaneous femoral	Percutaneous femoral and left axillary	Percutaneous femoral	Percutaneous femoral
Proximal component	Captivia; VAMF2824C150	Captivia; VAMF3834C150	Captivia; VAMF3430C150	Captivia; VAMF3030C150	Captivia; VAMF3430C150	Captivia VAMF3834C150
Distal bifurcated component	Endurant	Gore Excluder	Endurant	Endurant	Endurant	Endurant
Modification time, minutes	134	119	128	114	111	124
Fluoroscopy time, minutes	92	87	90	85	69	96
Operative time, minutes	200	235	180	412	190	210
Endograft profile, F	22	24	24	22	24	24
Iliac axis tortuosity index	1.2	1.0	1.1	1.3	1.4	1.1
Aortic paravisceral angle	22	26	19	20	16	21
Renal vessel caudal orientation >30°	–	–	–	RRA	–	–
Bridging stent used	iCover (iVascular)	VBX (Gore)	VBX (Gore)	iCover (iVascular)	iCover (iVascular)	iCover (iVascular)
Horizontal misalignment^a^	–	10° RRA	–	8° SMA	–	–
Any vertical misalignment (>5 mm)^a^	–	–	–	–	–	–

*RRA,* Right renal artery; *SMA,* superior mesenteric artery.

a Measured on the first postoperative computed tomography angiogram.

The mean time required for modification was 121 ± 18 minutes, and in no case was stent bending for adequate fenestration allocation required. During deployment of the visceral segment, five patients did not require additional main graft rotation; in one patient, a ∼10° adjustment was required.

The intraoperative final fenestration alignment on cone-beam CT demonstrated regular geometric conformation in all cases, and technical success was 100%. The mean length of stay was 4 ± 1 days, with two patients monitored in the intensive care unit for 2 days for cardiac and renal function assessment. No postoperative adverse events and no reintervention at 30 days were reported. The first postoperative CT angiogram demonstrated accurate vertical alignment in all cases and horizontal misalignment of <15° in two fenestrations.^12^ Within 3 months after the intervention, no type Ia/Ib or III, IV, or V endoleaks were identified. A type II endoleak with no sac expansion was identified in two patients. No structure lesions of the main body, reinforced fenestration, or bridging stent and no major adverse events were registered.

## Discussion

The use of PMEG represents a valid endovascular treatment option for complex AAAs presenting in the urgent setting and for asymptomatic patients but at high risk of imminent rupture that cannot wait for a custom-made device. In the subset of patients presenting with asymptomatic large complex AAAs or type IV TAAAs, it has been demonstrated how the use of PMEGs results in better outcomes in terms of reduced major adverse events compared with those presenting in the emergent setting (11% vs 25%), with increased technical success for type IV TAAAs compared with extent type I to III TAAAs (99.4% vs 97%).^8^

In the urgent setting, the other two main available options are parallel grafts or readily available off-the-shelf thoracoabdominal grafts. Comparing the PMEG to parallel grafting, Smith et al.^13^ demonstrated in a retrospective cohort study of >800 cases from the Vascular Quality Initiative a significantly reduced risk of postoperative complications with a dramatic decrease in critical gutter endoleaks in favor of PMEGs. Georgiadis et al.^14^ compared the use of off-the-shelf thoracoabdominal grafts with PMEG, demonstrating the safety and effectiveness in both elective and acute setting for complex AAAs. However, off-the-shelf devices have been developed for TAAAs, and their use for complex AAAs or type IV TAAAs requires extensive coverage of ≥11 to 12 cm above the celiac trunk compared with the 2.5 to 5 cm needed in the case of complex AAAs treated by PMEG. This could help in reducing the paraplegia risk, especially in urgent cases in which a complete and single-stage procedure is required to exclude the aneurysm.

The drawback of PMEGs is the extreme variability in the treated aneurysm extent, planning, modification technique, and use of fenestrations or branches. Melo et al.^8^ pointed how the data quality of PMEGs reported in the literature is low. Of >900 PMEGs, only one case series was based on the Valiant Captivia. A few sporadic cases used the abdominal Endurant graft (Medtronic), and the vast majority used Cook Medical devices (both thoracic and abdominal components). The indications for treatment ranged from extent type I, II, and III TAAAs to short neck AAAs, and single and multiple fenestrations or branches were used.

In this experience, we tried to standardize as much as possible the patient selection, measurement method, and PMEG technique of modification. We applied a four-fenestrated PMEG in all cases of complex AAAs, whether elective and urgent, and excluded extensive type I to III AAAs. This is because those long segment aneurysms are treated with long aortic coverage with a thoracic EVAR graft and, thus, representing a different grade of complexity and possible complications as previously reported in a large study review.^8^ Specifically, our coverage is based on applying a short length of coverage above the celiac trunk as suggested by the SVS guidelines,^9^ because low supraceliac coverage (<5 cm) represents that with the lowest paraplegia risk.^15^ In addition, the anatomical feasibility parameters defined and the standardized method of measurement for fenestration allocation allow for the clear selection of a subset of patients with no additional bias.

Regarding the type of graft used for modification, the vast majority in the literature are represented by the Cook Medical platform.^16^ Recently, Chait et al.^17^ reported the long-term outcomes of >150 Cook PMEGs, with excellent results in terms of early mortality, freedom from aortic-related mortality, and target instability, especially for complex AAAs compared with TAAAs.

To the best of our knowledge, only a single experience has been reported of the Valiant Captivia PMEG in the version without free flow.^18^ In this experience, the investigators reported 18 cases. After complete unsheathing, the graft was introduced in a sterile aortic three-dimensional model for precise fenestration allocation and then resheathed. We believe that leaving the graft on its device might reduce the risk of infection, reducing manipulation and contact with other materials, as the printed aortic model. The other two case reports using the Valiant platform for the treatment of the paravisceral segment are from Gibello et al,^19^ who used a Valiant Navion graft, and Joseph et al,^20^ who applied minicuff-augmented fenestration using the Valiant Captivia.

The Valiant Captivia represents a good option as a PMEG, because of the large distance between the stents, the simple resheathing, and the possibility to construct a diameter-reducing tie without alteration of the graft fabric. In particular, we selected the Valiant Captivia stent in the free-flow configuration because this allows for better proximal supraceliac wall apposition and fixation with minimal oversizing and the advantage of reducing the mismatch with the smaller pararenal aorta diameter. The other main reason we prefer the Captivia stent is that the stent design has a relatively large distance between the peaks (17-25 mm depending on graft diameter), allowing for easy fenestration allocation without the need for strut bending, such as in the case of a Gore graft (W.L. Gore & Associates) or Zenith TX2 graft (Cook Medical). This structural detail increases graft integrity with a lower risk of losing radial force at that level. The other graft with a large distance between peaks is the Zenith Alpha thoracic graft (18-19 mm).^21^ However, with this platform, the posterior constraining wire requires multiple needle punctures of the fabric. This is not needed with the Captivia platform, because the wire is passed straight through the tight knots in the stents and, thus, reducing the risk of a type IV endoleak. Finally, resheathing of the graft is easy and smooth, with good fenestration constraints into the shaft tube and no risk of graft alteration during this maneuver.

The modification of the 8-shaped marker into an “S” on top of the first stent at the 0° position has been developed to give a clear anteroposterior orientation, allowing for clear reference for adequate positioning and deployment from the beginning. Furthermore, reinforcement of the fenestration with the tip of the 0.018-in. guidewire allows for clear visualization of the fenestration and evaluation of the orientation of the circle in relation to the circle of the fusion imaging, improving the orientation. This is particularly helpful in understanding the need for minimal rotation during visceral deployment to guarantee correct orientation of the fenestrations. The Valiant platform has a 22F to 25F outer device profile, which could limit its applicability in cases of small diameter iliofemoral access. Also, in cases of tortuous access, the flexible shaft can lose graft rotational control during deployment, requiring advancement over an introducer sheath.^12^

The adjunct of preloaded wires has already been reported in several experiences of TAAA repair, especially in the case of branched PMEGs, with the advantage of facilitating target vessel cannulation in the case of complex aortic or renal–visceral anatomy.^22^ In our experience, in which only juxtarenal and pararenal AAAs and type IV TAAAs were selected, the addition of modified branches is not required, and fenestrations in general have a limited need for preloading wires. Also, the recent development of dedicated steerable sheaths can facilitate vessel and fenestration catheterization. In this experience, preloaded wires were not applied to the PMEGs to limit cumbersome additional steps to modification. To the best of our knowledge, this is the first case series using a dedicated Valiant Captivia graft with free flow as a PMEG for the paravisceral segment. The results obtained with this small number of cases are encouraging and might indicate that this technique protocol is feasible, safe, and reproducible. Our future perspective is to collect multicenter data with a larger number of patients and longer follow-up to validate a reproducible and effective standardized method for complex AAA repair when four-fenestrated PMEG use is required.

## Conclusions

To the best of our knowledge, this represents the first attempt to standardize a protocol for PMEG usage. This effort will allow for a clear understanding of whether this method is valid and effective, with limited variables related to eventual interoperator differences on anatomical feasibility and the technique for graft modification. It will serve as a benchmark for comparison with previously reported experiences or future additional standardized methods. A larger number of cases are needed to demonstrate midterm outcomes.

## Disclosures

Michele Piazza: Advisory Board and Proctor for Medtronic. All fees paid to Department of Cardiac, Thoracic, Vascular Sciences and Public Health.

## Appendix

Supplementary Video Step by step standardized technique for four-fenestrated physician-modified endograft (PMEG).
